# Editorial: Machine Learning-Based Methods for RNA Data Analysis

**DOI:** 10.3389/fgene.2022.828575

**Published:** 2022-05-25

**Authors:** Lihong Peng, Jialiang Yang, Minxian Wang, Liqian Zhou

**Affiliations:** ^1^ College of Life Sciences and Chemistry, Hunan University of Technology, Zhuzhou, China; ^2^ School of Computer, Hunan University of Technology, Zhuzhou, China; ^3^ Geneis (Beijing) Co. Ltd., Beijing, China; ^4^ CAS Key Laboratory of Genome Sciences and Information, Beijing Institute of Genomics, Chinese Academy of Sciences, Beijing, China; ^5^ University of Chinese Academy of Sciences, Beijing, China

**Keywords:** machine learning, lncRNA, circRNA, microRNA, mRNA, gene expression

RNA is a type of extremely important biological macromolecules, which play key roles in all aspects of life activities and biological processes through its interactions with other biological entities Wang et al. (2021); Zhang et al. (2021). Thus, it is critical to identify complex biological associations between RNA and other biological entities Mu et al. (2020); Deng et al. (2018). Although experimental methods have been applied to analyze RNA data, especially identify various associations between RNA molecules and complex diseases, they are usually time-consuming and resource demanding. Machine learning aims to simulate human learning ways in real time and divide the existing content into knowledge structures to advance learning efficiency. It can effectively use available electronic data to boost learning performance or implement accurate prediction Mohri et al. (2018). Furthermore, it still improves more evidence-based decision-making in the area of life science Jordan and Mitchell (2015). With the advancement of next generation sequencing techniques, machine learning-based methods discovered a large number of useful information from abundant RNA data and thus provide an effective way for the analysis of RNA data. Consequently, through machine learning techniques, we can design powerful models and algorithms to discovery diverse associations between RNA molecules themselves (such as microRNAs, mRNA, circular RNAs, and long noncoding RNAs) and between RNA molecules and complex diseases. We can further infer novel molecular markers for diagnosis and prognosis of corresponding diseases based on the identified associations.

Based on the assumption of “guilt-by-association” and machine learning technologies, accumulated computational methods have been developed to analyze RNA data Liu et al. (2020); Chu et al. (2021). However, the performance of most methods remains unsatisfying due to data complexity and heterogeneity. Therefore, this research topic serves as a forum to develop new machine learning algorithms to improve RNA data analyses.

MicroRNAs (miRNAs) are a class of short and endogenous noncoding RNAs Wang et al. (2020); Chen et al. (2019a). miRNAs can control gene expression based on translational repression or messenger RNA (mRNA) degradation and exhibit strong associations with a variety of disease including neurodegenerative diseases and cancers Saliminejad et al. (2019). Chen et al. designed a few representative machine learning-based algorithms to identify potential microRNA-disease associations Chen et al. (2018, 2019b).

To find robust biomarkers associated with prostate cancer, Ning et al. designed a multi-omics data fusion method by integrating directed random walk and Support Vector Machine (SVM). They compared their proposed pathway-based method with five other methods including the Median method, Mean method, component analysis method, pathway activity inference method based on condition-responsive gene analysis, and directed random walk method. The results from cross validation showed that their proposed method computed the best average AUC and accuracy in three within-datasets and other 10 cancer datasets. They inferred that hsa-miR-106b and hsa-miR-20b may be the shared miRNA-mediated subpathway biomarkers in GSE21036, GSE14794, and “PRAD-TCGA” datasets.

The inference of cancer-related circulating biomarkers has become one of the most important research directions on clinical cancer diagnosis. To identify extracellular microRNAs for pan-cancers, Yuan et al. integrated Boruta feature filtering, max-relevance and min-redundancy feature selection, incremental feature selection, synthetic minority oversampling method, and four classification models including random forest, SVM, *k*-nearest neighbors, and decision trees. They conducted 10-fold cross validation for 20 times. The results showed that SVM obtained better accuracies and Matthew correlation coefficients compared to other three black-box classifiers. They predicted that hsa-miR-5100 and hsa-miR-6088 have strong associations with pan-cancer.

Most of chronic liver diseases are caused by non-alcoholic fatty liver diseases (NAFLD). To capture miRNAs, circRNAs, and genes associated with NAFLD, Du et al. built a circRNA-miRNA-mRNA network and provided a novel perspective for the inhibition and therapy of NAFLD using functional enrichment analysis and protein interaction network analysis. They found that the crosstalk between hsa_circ_000031, miR-6512-3p, and PEG10 may participate in NAFLD’s pathogenesis and the crosstalk could be the underlying biomarkers of NAFLD.

mRNA stability affects gene expression in almost all organisms from bacteria to human. Clinical trials have revealed that mRNA vaccines provide a safe and effective immune response in human Zhang et al. (2019). Therefore, mRNA vaccines exhibit a powerful alternative to traditional vaccine approaches Pardi et al. (2018). To discover potential biomarkers associated with cutaneous melanoma, Bai et al. combined univariable Cox proportional hazards regression and random survival forest algorithm and designed a four-mRNA signature approach. The proposed four-mRNA signature method was compared with two clinical prognostic markers (melanoma clark level and tumor stage) and obtained the best AUC and sensitivity. They found the four-mRNA signature (CD276, UQCRFS1, HAPLN3, and PIP4P1) could be a prognostic signature for cutaneous melanoma patients.

Circular RNAs (circRNAs) are a class of RNAs with covalently closed structure and high stability Vo et al. (2019). circRNAs can serve as a new strategy for diagnosis and treatment of diseases Zeng et al. (2020). Autism is a multifactorial neurodevelopmental disease and usually involves in mental disorder, attention deficit, and intellectual disability. To analyze circRNA expression in autism in the mouse brain, Wang et al. built a circRNA-based competing endogenous RNA network. They successfully established a mouse autism model and measured repetitive self-grooming behaviors. Furthermore, they constructed a circRNA-miRNA-mRNA network composed of 1,059 circRNAs, 1,926 miRNAs, and 6,730 mRNA. Third, they performed gene ontology and pathway enrichment analysis and statistical analysis. Finally, they identified 1,059 differentially expression circRNAs associated to autism.

Long noncoding RNAs (lncRNAs) are a class of noncoding RNAs involved in diverse biological processes Peng et al. (2022); Jia and Luan (2022). lncRNAs are aberrantly expressed in numerous cancers and demonstrate crucial roles in oncogenic and tumor suppressive activities Zhou et al. (2021); Liu et al. (2022). lncRNAs implement their biological functions by linking to RNA-binding proteins. Deep learning-based methods, such as LPIDF Tian et al. (2021), deep forest Wang et al. (2021), Capsule-LPI Li et al. (2021), and LPI-DLDN Peng et al. (2021), were widely applied to detect lncRNA-protein interactions. Breast cancer is one of the most common malignant tumors and causes the leading mortality in women. To identify immune-associated lncRNAs for breast cancer prognosis, Huang et al. designed a novel framework to identify immune lncRNA signatures as prognostic marker for breast cancer combining Cox regression analysis and iterative Lasso Cox regression analysis. The proposed model was validated the performance in two independent cohorts by comparing with known prognostic biomarkers and obtained an AUC of 0.86. The results showed that the proposed model can effectively analyze immune-associated lncRNAs for breast cancers based on ROC analysis, Kaplan-Meier analysis, univariate and multivariate Cox regression analysis, gene set enrichment analysis, and gene set variation analysis. Furthermore, they confirmed that lncRNA signatures could independently assess breast cancer survival.

Gene expression analyses contribute to prioritizing potential disease genes and identifying transcriptional regulatory programmes Toro-Domínguez et al. (2021). With the development of single-cell RNA sequencing technologies, a large number of machine learning-related models and algorithms are increasingly exploited to analyze single-cell RNA sequencing data Zhu et al. (2022); Xu et al. (2020). Moni et al. Mohri et al. (2018) conducted a large number of comparative genomic and transcriptomic analyses to capture key gene expression pathways associated with SARS-CoV-2.

To accurately impute gene expression information for multiple tissue types with minimal reconstruction error, Vinas et al. developed two deep learning models, pseudo-mask imputer and generative adversarial imputation network-based method. They compared their proposed methods with several state-of-the-art imputation methods on RNA-seq data from the GTEx project. The results showed that pseudo-mask imputer outperformed all other methods in inductive imputation and generative adversarial imputation network-based method obtained the highest performance in in-place imputation in terms of the coefficient of determination and runtime. They observed that several genes (such as PSMB6, COX6C, PSMD7 and PSMA2) exhibited different distributions in the Alzheimer’s disease pathway.

Soft tissue sarcoma is a type of tumors accounting for 1% in adult cancers. To precisely observe new biomarkers and therapeutic targets for the disease, Liu et al. used risk characteristics and transcriptome data and built a risk signature and nomograms for patients with soft tissue sarcoma based on glycolysis-related genes. The results demonstrated that the proposed model computed the best AUCs. They screened seven glycolysis-related genes associated with soft tissue sarcoma.

Dysregulation of alternative splicing is very important to tumorigenesis and microenvironment formation. To predict splicing factors related to alternative splicing in breast cancer, Deng et al. performed genome-wide analysis of the alternative splicing events in breast cancers based on differential and prognostic analyses. The proposed method computed relatively lower false discovery rate. They detected a few differentially expressed alternative splicing events and independent prognostic factors associated with breast cancer.

Pathological neovascularization in choroid is a major cause of blindness. To capture key signaling pathways in choroidal neovascularization, Jia et al. first performed three bioinformatics analyses, which include hierarchical cluster analysis, weighted gene co-expression network analysis, and protein-protein interaction network analysis. They then implemented hematoxylin and eosin staining, CD31 immunohistochemistry, and reverse transcription quantitative PCR. The results showed that differentially expressed genes in chroid were mainly linked to membrane transport.

RNA molecules have close linages with various diseases. The inference of diverse associations between RNAs and diseases contributes to revealing the pathogenic mechanism of complex diseases and investigating corresponding biomarkers, and further designing appropriate therapeutic strategies. On the research topic, researchers designed various machine learning-based methods and used multiple bioinformatics tools to analyze diverse RAN molecules. They obtained relatively better results and found a few biomarkers. We hope that the topic could improve RNA analyses and promote the diagnosis and treatment of related diseases.
